# Exploring risk factors for cervical lymph node metastasis in papillary thyroid microcarcinoma: construction of a novel population-based predictive model

**DOI:** 10.1186/s12902-022-01186-1

**Published:** 2022-11-04

**Authors:** Yanling Huang, Yaqian Mao, Lizhen Xu, Junping Wen, Gang Chen

**Affiliations:** 1 Shengli Clinical Medical College of Fujian Medical University, Fuzhou, China; 2 Department of Endocrinology, Zhongshan Hospital Xiamen University, Xiamen, China; 3 Department of Internal Medicine, Fujian Provincial Hospital Jinshan Branch, Shengli Clinical Medical College of Fujian Medical University, Fuzhou, China; 4 Department of Endocrinology, Fujian Provincial Hospital, Shengli Clinical Medical College of Fujian Medical University, Fuzhou, 350001 China

**Keywords:** Papillary thyroid microcarcinoma cervical lymph node metastasis, Machine learning, Conventional regression model, Risk factors, Prediction model

## Abstract

**Background:**

Machine learning was a highly effective tool in model construction. We aim to establish a machine learning-based predictive model for predicting the cervical lymph node metastasis (LNM) in papillary thyroid microcarcinoma (PTMC).

**Methods:**

We obtained data on PTMC from the SEER database, including 10 demographic and clinicopathological characteristics. Univariate and multivariate logistic regression (LR) analyses were applied to screen the risk factors for cervical LNM in PTMC. Risk factors with *P* < 0.05 in multivariate LR analysis were used as modeling variables. Five different machine learning (ML) algorithms including extreme gradient boosting (XGBoost), random forest (RF), adaptive boosting (AdaBoost), gaussian naive bayes (GNB) and multi-layer perceptron (MLP) and traditional regression analysis were used to construct the prediction model. Finally, the area under the receiver operating characteristic (AUROC) curve was used to compare the model performance.

**Results:**

Through univariate and multivariate LR analysis, we screened out 9 independent risk factors most closely associated with cervical LNM in PTMC, including age, sex, race, marital status, region, histology, tumor size, and extrathyroidal extension (ETE) and multifocality. We used these risk factors to build an ML prediction model, in which the AUROC value of the XGBoost algorithm was higher than the other 4 ML algorithms and was the best ML model. We optimized the XGBoost algorithm through 10-fold cross-validation, and its best performance on the training set (AUROC: 0.809, 95%CI 0.800–0.818) was better than traditional LR analysis (AUROC: 0.780, 95%CI 0.772–0.787).

**Conclusions:**

ML algorithms have good predictive performance, especially the XGBoost algorithm. With the continuous development of artificial intelligence, ML algorithms have broad prospects in clinical prognosis prediction.

**Supplementary Information:**

The online version contains supplementary material available at 10.1186/s12902-022-01186-1.

## Background

Over the past few decades, the incidence of thyroid cancer has been increasing. Among which papillary thyroid microcarcinoma (PTMC) accounted for a large proportion [1].PTMC was defined as a papillary thyroid carcinoma (PTC) with a maximum diameter of 1 cm or less. Although most PTMC incidences appear indolent [2], a small number of cases still show significant biological aggressiveness, such as early metastasis and lymph node involvement [3]. Cervical lymph node metastasis (LNM), especially in the central compartment, were found in many patients with PTC. Some studies showed that it could be found in 29.4–51.3% of PTC [4, 5], and was associated with an increased risk of local recurrence and mortality [6]. Accurate preoperative identification of cervical LNM was essential for clinical management. Many clinical institutions had used their clinical data to analyze the risk factors and constructed models for cervical LNM of PTMC. However, these models were mostly established based on a small sample size, thus have certain limitation in predicting outcome [5, 7, 8]. There were also some predictive models constructed by conventional statistics based on the SEER database, which had a large number of clinical data of patients with PTMC [9, 10]. But at present it appeared that these models often utilize conventional regression-based approaches and fail to properly consider the nonlinear relations and interactive effects, thus may not provide optimal prediction power.

In recent years, advancements in the field of artificial intelligence had introduced machine learning (ML) as a highly effective tool in many eras including medical research. ML was less concerned with model interpretability and more mathematically focused on predictive performance and model generalization around cross-validation and iterative improvement of algorithms. ML can deal with a large number of variables that might have nonlinear and higher-dimensional relationships. For these reasons, ML often out-performance explanatory models when complex, high-dimensional data was available. As of today, ML methods are widely applied in the medical field including image interpretation, pathological diagnosis, risk factor screening, etc. [11–14]. Here we aim to establish a machine learning-based predictive model for predicting the cervical LNM in PTMC patients. The goal was to identify important risk factors in cervical LNM of PTMC and provide clinicians with more personalized clinical decision in the management of the PTMC.

## Methods

### Data sources and study population

**Fig. 1:**
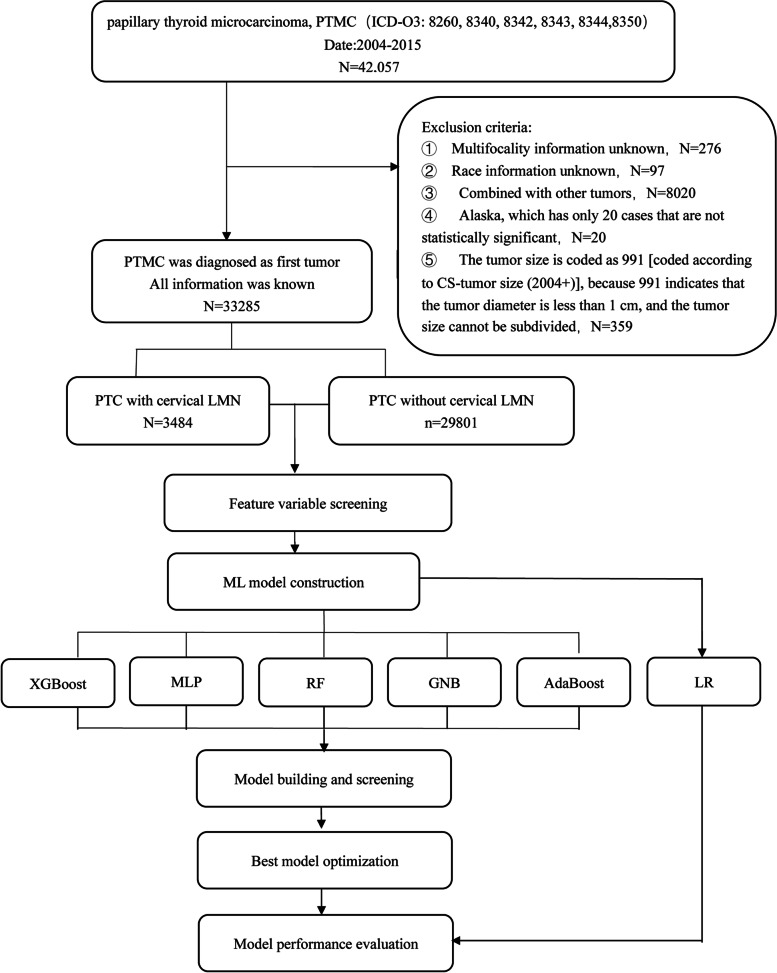
Flow chart of patients selection and study design

Data was obtained from the SEER database. We used SEER*stat 8.3.9 software for data acquisition. The subjects of the study were patients diagnosed with papillary thyroid microcarcinoma from 2004 to 2015 in 18 regions of the United States. Inclusion criteria: ① There was no restriction on age and gender. ② The histological type was papillary thyroid. ③ The tumor size≤10 mm.Exclusion criteria: ① Unknown information/not applicable. ② PTMC was not the only tumor (that is, combined with other tumors). The detailed research process was shown in Fig. 1. This study does not require institutional review board approval because it involves the use of publicly available data.

### Data selection and definition

We extracted information from the SEER database according to the SEER User Guide and the Collaborative Data Collection System (CS Handbook Online Help) [15]. Finally, we selected demographic characteristics that were available in the SEER database, including age, gender, race, region of residence, marital status, and clinicopathological characteristics, including tumor size, tumor pathological type, multifocality, and extrathyroidal extension. Detailed definitions and classifications of variables can be found in Supplementary Materials Table S1. Data extraction, definition, and classification were conducted by two of the authors (Yanling Huang and Yaqian Mao), Any resulting discrepancies were resolved by discussion.

### Risk factor screening and conventional regression model construction

In the baseline analysis, we performed correlation tests between variables by Pearson correlation analysis and presented the results in the form of heat maps. We used variance inflation factor (VIF) [16] to assess multicollinearity among variables. In the univariate LR analysis, variables with *P* < 0.05 were selected as the related risk factors for cervical LNM and were included in the multivariate analysis. 95% confidence intervals (CIs) and odds ratios (ORs) for risk factors were calculated using multivariate LR analysis with forward stepwise regression. Variables with *P*<0.05 in multivariate LR analysis were selected as modeling variables to construct the conventional regression model.

We used forest plots to show OR values ​​and 95% CIs of statistically significant factors. We visualized conventional regression analysis in the form of a nomogram. Each predictor variable was assigned to a point in the nomogram. By adding the scores for each variable, ranging from 0 to 100, we can predict the probability of developing cervical LNM in a given patient.

### Machine learning model construction and evaluation

Similarly, we used the variables screened by univariate analysis and multivariate analysis as modeling variables for ML. All data were randomly divided into training set and validation set in a ratio of 8:2. Five commonly used machine learning algorithms were applied in the training set respectively, including gaussian naive bayes (GNB), extreme gradient boosting (XGBoost), random forest (RF), multi-layer perceptron (MLP) and adaptive boosting (AdaBoost). GNB is a variant of Naïve Bayes, which is a supervised machine learning classification algorithm based on the Bayes theorem. Various strengths of GNB are its convenience, computation speed, scalability with small data and flexibility with continuous and discrete features [17]. XGboost is an efficient and scalable machine learning classifier based on the Gradient Boosting Decision Tree (GBDT) algorithm. It provides parallel tree boosting and enhances performance by using learning rate, subsampling ratio, and maximum tree depth to make the model less prone to overfitting [18]. RF algorithm is a combined classifier algorithm based on cart decision tree, which allows construction of multiple tree classification models. Although they are strong in modeling capacity, tree based models tended to have some overfitting of the training set, thus the used for generalization might be limited [19]. MLP is a feedforward artificial neural network. The characteristics of MLPs include multiple layers and nonlinear activation for nodes of hidden and output layers, which enabled the algorithms to deal with nonlinear data [20]. AdaBoost is a typical boosting algorithm. By reducing the classification error of individual learner each time, the importance of good individual learner is increased, and the final integrated learner is obtained. Adaboost is considered more effective at handling an unbalanced dataset than Random Forests [21].

In the construction of the ML model, resampling method was adopted to obtain the best modeling parameters and tuning was considered for ML-based models to avoid overfitting. Model evaluation was performed using area under the receiver operating characteristic (AUROC) curve, accuracy, sensitivity and specificity. The model with the largest AUROC was selected for further optimization. Finally, a 10-fold cross-validation was performed on the selected model to improve the accuracy. Performance of the model was evaluated by the test set, thereby establishing the optimal ML model. We used XGBoost’s own algorithm to rank variable importance. SHapley Additive exPlanations (SHAP) was applied to provide an explanation for our predictive model [22].

### Performance evaluation and comparison

We evaluated the performance of the conventional regression model and ML model through the AUROC, sensitivity, specificity, accuracy, and negative predictive value.

### Statistical analysis

All statistical analyses were performed using R version 3.6.3 (http://www.r-project.org/) and Python version 3.7.0 (https://www.python.org/downloads/release/python-370/) Categorical variables were expressed using frequencies and percentages, and baseline characteristic analysis was performed by chi-square test. The Python packages “scikit-learn==0.22.1”, “Xgboost==1.2.1”, “Lightgbm==3.2.1” were used for ML algorithms. All statistical analyses were performed with a two-sided test, with a *P* value less than 0.05 indicating a significant difference.

## Results

### Demographics features

A total of 33,285 patients were included in the final data analysis. Of these patients, 3484 had cervical LNM (10.5%), including patients with central and lateral LNM. The flowchart of study was shown in Fig. 1. The baseline characteristics of all PTMC patients were shown in Table 1.

**Table 1:** Characteristics of patients of PTMC with cervical LMN identified from the SEER database

Characteristic	Total (***n*** = 33,285)	Cervical LMN (−) (***n*** = 29,801)	Cervical LMN (+) (***n*** = 3484)	***p***-value
**Age, n(%)**				< 0.001
<25	1059 (3.182)	799 (2.681)	260 (7.463)	
25–39	7062 (21.217)	6004 (20.147)	1058 (30.367)	
40–54	13,173 (39.576)	11,849 (39.760)	1324 (38.002)	
55–69	9552 (28.698)	8863 (29.741)	689 (19.776)	
≥ 70	2439 (7.328)	2286 (7.671)	153 (4.392)	
**Gender, n(%)**				< 0.001
Female	27,569 (82.827)	25,083 (84.168)	2486 (71.355)	
Male	5716 (17.173)	4718 (15.832)	998 (28.645)	
**Race, n(%)**				< 0.001
White	27,796 (83.509)	24,800 (83.219)	2996 (85.993)	
Black	2245 (6.745)	2147 (7.204)	98 (2.813)	
Other^a^	3244 (9.746)	2854 (9.577)	390 (11.194)	
**Marital status, n(%)**				< 0.001
Married	22,671 (68.112)	20,383 (68.397)	2288 (65.672)	
Single	6217 (18.678)	5349 (17.949)	868 (24.914)	
Divorced	2591 (7.784)	2369 (7.949)	222 (6.372)	
Widowed	1479 (4.443)	1399 (4.694)	80 (2.296)	
Separated	279 (0.838)	258 (0.866)	21 (0.603)	
Partner	48 (0.144)	43 (0.144)	5 (0.144)	
**Region, n(%)**				< 0.001
East	14,013 (42.100)	12,724 (42.697)	1289 (36.998)	
Pacific Coast	14,235 (42.767)	12,544 (42.093)	1691 (48.536)	
Northern Plains	2734 (8.214)	2467 (8.278)	267 (7.664)	
Southwest	2303 (6.919)	2066 (6.933)	237 (6.803)	
**Histology, n(%)**				< 0.001
Classical type	23,131 (69.494)	20,404 (68.468)	2727 (78.272)	
Follicular variant	9652 (28.998)	8986 (30.153)	666 (19.116)	
Oxyphilic variant	42 (0.126)	36 (0.121)	6 (0.172)	
Encapsulated variant	183 (0.550)	177 (0.594)	6 (0.172)	
Tall-cell variant	192 (0.577)	131 (0.440)	61 (1.751)	
Diffuse sclerosing variant	85 (0.255)	67 (0.225)	18 (0.517)	
**Tumorsize, n(%)**				< 0.001
<5 mm	17,298 (51.969)	16,377 (54.955)	921 (26.435)	
≥ 5 mm	15,987 (48.031)	13,424 (45.045)	2563 (73.565)	
**ETE, n(%)**				< 0.001
No	31,288 (94.000)	28,585 (95.920)	2703 (77.583)	
Yes	1997 (6.000)	1216 (4.080)	781 (22.417)	
**Multifocality, n(%)**				< 0.001
Solitary tumor	21,618 (64.948)	20,174 (67.696)	1444 (41.447)	
Multifocal tumor	11,667 (35.052)	9627 (32.304)	2040 (58.553)	
**Laterality, n(%)**				0.732
Unilateral	33,144 (99.576)	29,676 (99.581)	3468 (99.541)	
Bilateral	141 (0.424)	125 (0.419)	16 (0.459)	

*Note*: ^a^Others include American Indian / Alaska native, Asian or Pacific islander

*Abbreviations*: *PTMC* Papillary thyroid microcarcinoma, *LNM* lymph node metastasis, *ETE* Extrathyroidal extension

### Univariate and multivariate logistic regression analyses

**Fig. 2:**
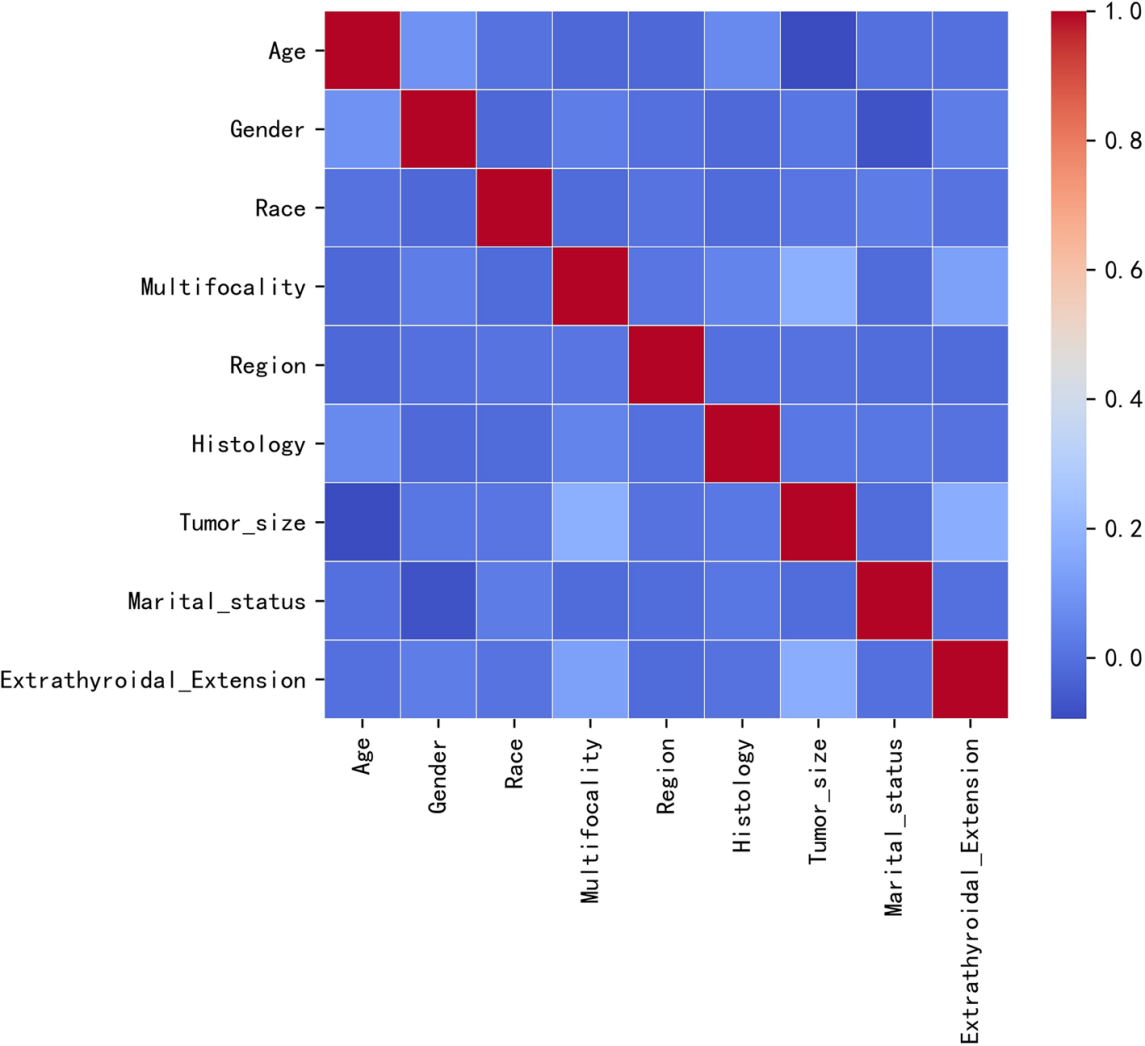
Pearson correlation test for variables

To explore the influence of variables on lymph node metastasis, a baseline analysis was carried out first, See Table 1 for the baseline analysis. Pearson correlation test was performed between all variables, and the correlation heat map shown that there was no significant correlation between them (Fig. 2). The inflation factor (VIF) of all variables was<10, indicating that there was no multicollinearity between the variables. Table 2 showed the results of univariate and multivariate LR of cervical LNM. In univariate LR analysis cervical LNM was significantly correlated with tumor size, ETE, multifocality, histology, region, marital status, race, gender and age (*P* < 0.001). The above mentioned risk predictors were all incorporated into the multivariate LR analysis. Multivariate LR indicated that among the demographic characteristics, male gender (OR: 2.279, 95%CI:2.086–2.488), single marital status (OR:1.152, 95% CI:1.042,1.273), people lived in Pacific Coast (OR:1.309, 95% CI:1.203–1.425) and Southwest residence (OR:1.171, 95% CI:0.999–1.367) were independent positive predictors of cervical LNM. While older age 25–39 years (OR:0.586, 95% CI: 0.492–0.7); 40–54 years (OR:0.362,95% CI:0.304–0.433); 50–69 years (OR:0.26, 95%C:0.215–0.314); ≥70 years (OR:0.239, 95%CI:0.186–0.306) were independent negative predictors of cervical LNM. For clinicopathological characteristics, compared with classical papillary thyroid microcarcinoma, the histology of the follicular variant (OR: 0.26, 95%CI: 0.215–0.314) and being encapsulated variant (OR: 0.316, 95%CI: 0.123–0.661) were negative predictors. While having columnar-cell/tall-cell variant (OR: 2.002, 95%CI: 1.405–2.825) was a positive predictor. Tumor size≥5 mm (OR: 3.634, 95%CI: 3.328–3.969), ETE (OR: 4.583, 95%CI: 4.115–5.103), and multifocal tumor (OR:2.359, 95%CI: 2.184–2.547) were all positive predictors for cervical LNM.

**Table 2:** Univariate and multivariate logistic regression analyses of cervical LNM

Characteristics	Univariate Analysis			Multivariate Analysis		
	OR	95%CI	*P*-Value	OR	95%CI	*P*-value
**Age**, No.(%)						
< 25	1	[Reference]	NA	1	[Reference]	NA
25–39	0.542	[0.464,0.632]	< 0.001	0.586	[0.492–0.7]	< 0.001
40–54	0.343	[0.295,0.399]	< 0.001	0.362	[0.304–0.433]	< 0.001
55–69	0.239	[0.204,0.280]	< 0.001	0.26	[0.215–0.314]	< 0.001
≥ 70	0.206	[0.166,0.255]	< 0.001	0.239	[0.186–0.306]	< 0.001
**Gender**, No.(%)						
Female	1	[Reference]	NA	1	[Reference]	NA
Male	2.134	[1.971,2.311]	< 0.001	2.279	[2.086–2.488]	< 0.001
**Race**, No.(%)						
White	1	[Reference]	NA	1	[Reference]	NA
Black	0.378	[0.308,0.464]	<0.001	0.551	[0.442–0.68]	< 0.001
Other^a^	1.131	[1.011,1.266]	0.032	0.906	[0.799–1.025]	0.119
**Marital status**, No.(%)						
Married	1	[Reference]	NA	1	[Reference]	NA
Single	1.446	[1.330,1.572]	< 0.001	1.152	[1.042–1.273]	0.006
Divorced	0.835	[0.723,0.964]	0.014	1.05	[0.898–1.223]	0.533
Widowed	0.509	[0.405,0.641]	< 0.001	0.885	[0.684–1.131]	0.339
Separated	0.725	[0.464,1.134]	0.159	0.646	[0.39–1.016]	0.072
Partner	1.036	[0.410,2.618]	0.941	0.782	[0.251–1.982]	0.636
**Region**, No.(%)						
East	1	[Reference]	NA	1	[Reference]	NA
Pacific Coast	1.331	[1.233,1.437]	< 0.001	1.309	[1.203–1.425]	< 0.001
Northern Plains	1.068	[0.930,1.227]	0.35	1.124	[0.966–1.303]	0.126
Southwest	1.132	[0.978,1.311]	0.095	1.171	[0.999–1.367]	0.048
**Histology**, No.(%)						
Classical type	1	[Reference]	NA	1	[Reference]	NA
Follicular variant	0.555	[0.508,0.606]	< 0.001	0.552	[0.502–0.606]	< 0.001
Oxyphilic variant	1.247	[0.525,2.962]	0.617	1.299	[0.472–3.019]	0.574
Encapsulated variant	0.254	[0.112,0.573]	0.001	0.316	[0.123–0.661]	0.006
Tall-cell variant	3.484	[2.565,4.733]	< 0.001	2.002	[1.405–2.825]	< 0.001
Diffuse sclerosing variant	2.01	[1.193,3.387]	0.009	1.472	[0.802–2.581]	0.193
**Tumor size**, No.(%)						
<5 mm	1	[Reference]	NA	1	[Reference]	NA
≥ 5 mm	3.395	[3.138,3.673]	< 0.001	3.634	[3.328–3.969]	< 0.001
**Extrathyroidal extension**, No.(%)						
No	1	[Reference]	NA	1	[Reference]	NA
Yes	6.792	[6.157,7.493]	< 0.001	4.583	[4.115–5.103]	< 0.001
**Multifocality**, No.(%)						
Solitary tumor	1	[Reference]	NA	1	[Reference]	NA
Multifocal tumor	2.96	[2.756,3.180]	< 0.001	2.359	[2.184–2.547]	< 0.001
**Laterality. No.(%)**						
Unilateral	1	[Reference]	NA			
Bilateral	1.095	[0.650,1.845]	0.732			

*Note*: ^a^Other include American Indian /Alaska native, Asian or Pacific islander;

*Abbreviations*: *PTMC* papillary thyroid microcarcinoma, *LMN* lymph node metastasis, *OR* Odds ratio, *CI* Confidence interval

### Performance of conventional regression model

**Fig. 3:**
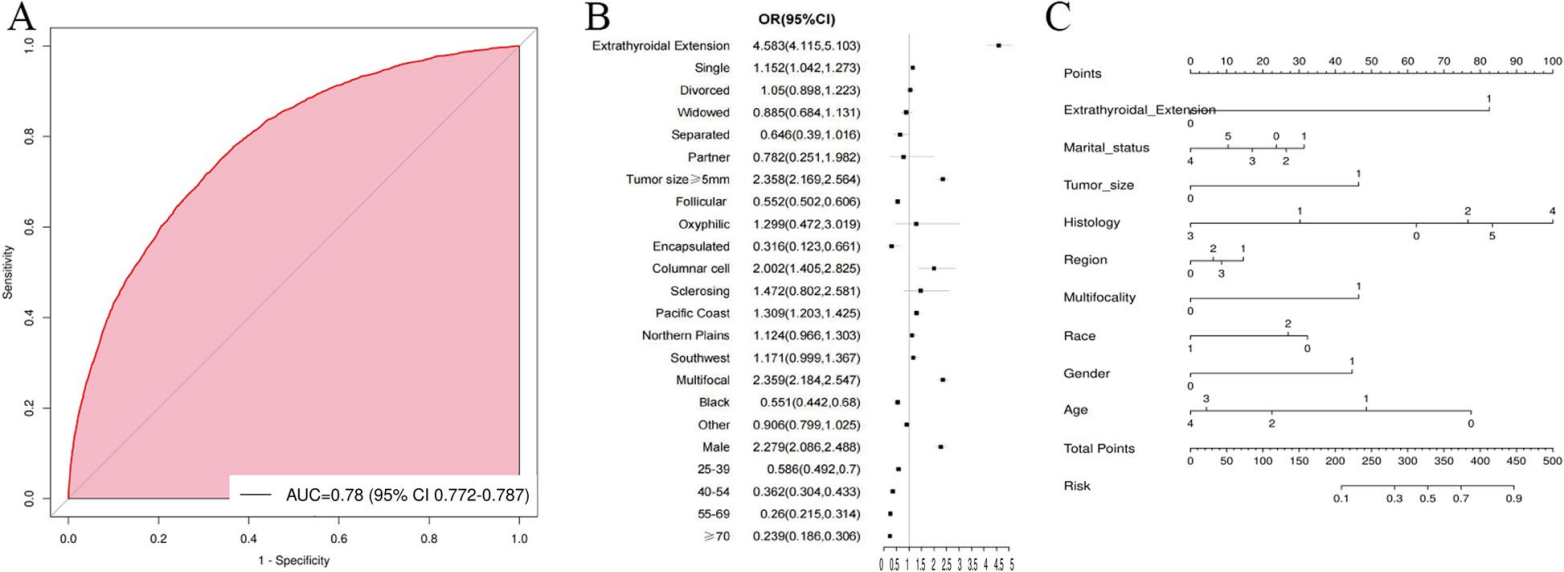
ROC curves, forest plot and nomogram of the LR model for cervical LNM in PTMC. Note: **A** shows the ROC curves of the multivariate LR. **B** shows the forest plot of the multivariate LR model. **C** shows the risk nomogram of the multivariate LR model

In the conventional LR model, the sensitivity of the model was 0.78, and the specificity was 0.718，demonstrated in Fig. 3A. Figure 3B was the forest plot of the conventional regression model. Figure 3C showed visualization of the results of the multivariate LR model in the form of nomogram.

### Machine learning model construction and screening

**Fig. 4:**
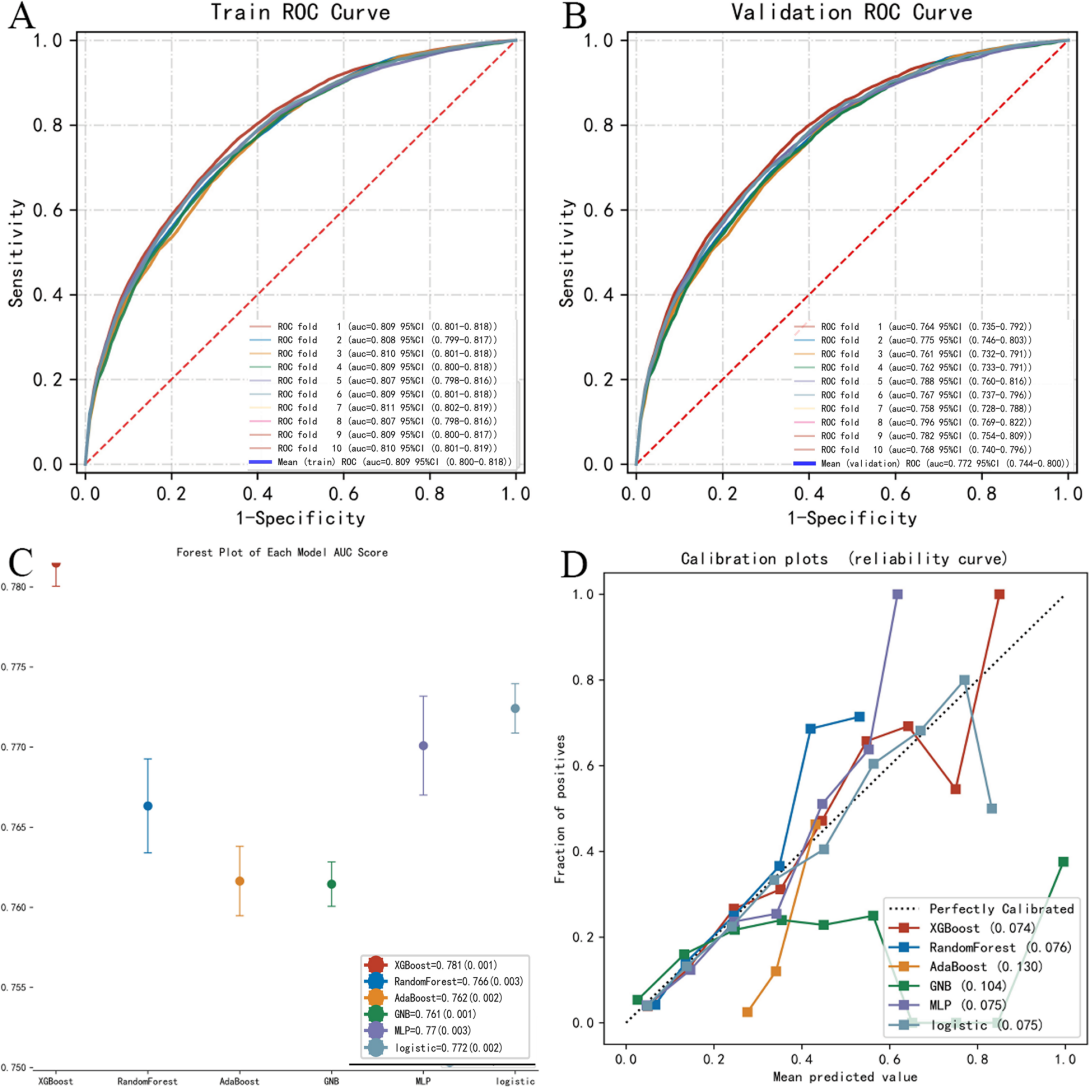
Model performance evaluation of different ML methods. Note: **A** showed the ROC curve of 5 different ML models in training set; **B** showed the ROC curve of 5 different ML models in validation set; **C** showed the AUC score forest plot of each model; **D** showed the reliability curve of each model

**Fig. 5:**
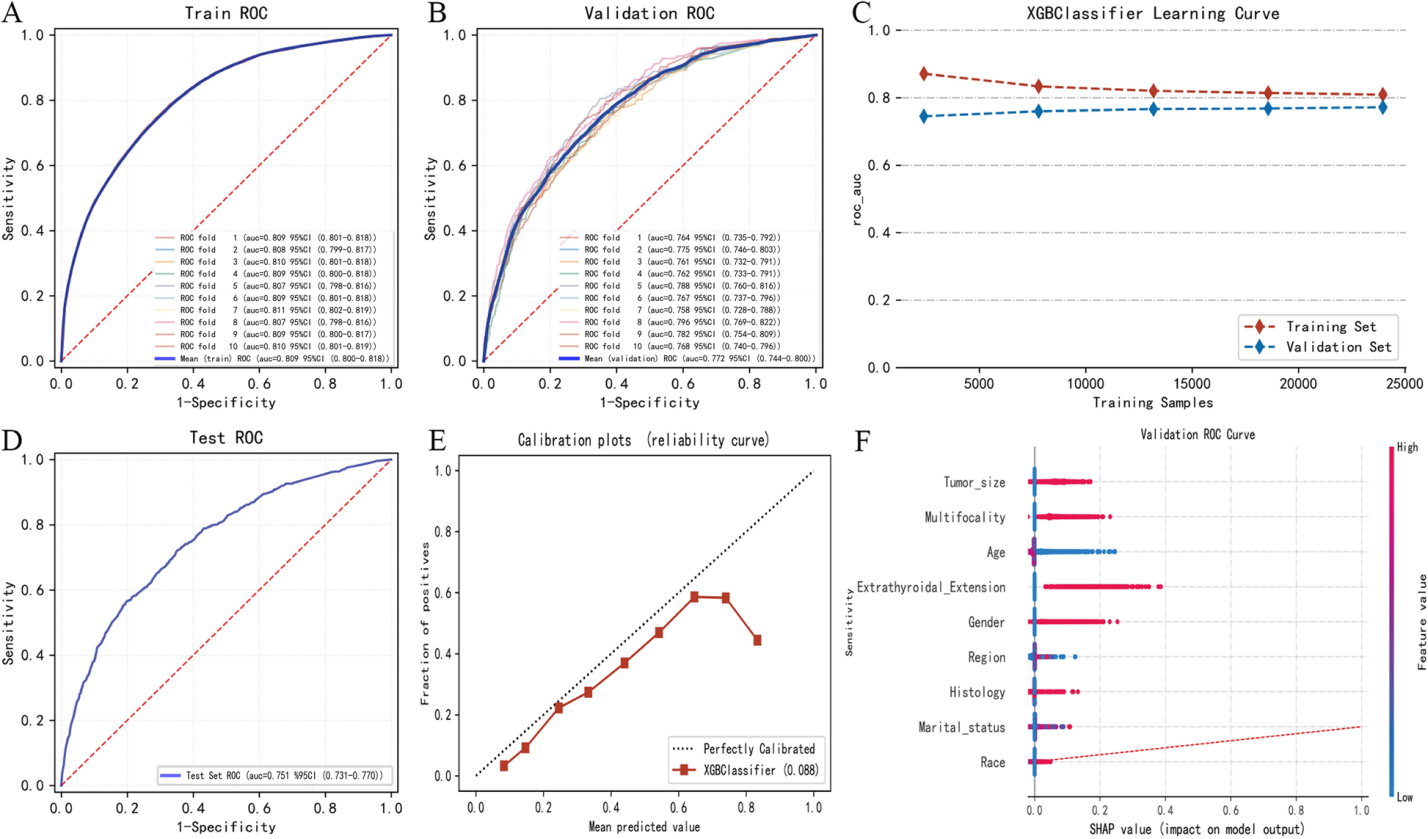
Optimization and visualization of the XGBoost model. Note: **A**, **B** and **D** displayed the ROC curve of the train, validation and test of the XGBoost model by 10-fold cross-validation. **C** showed the learning curve of the XGBoost classifier. **E** showed the reliability curve of XGBoost model. **F** showed the summary plots of SHAP values for the XGBoost model. For each feature, one point corresponds to a single patient. A point’s position along the x axis represented the impact that feature had on the model’s output for that specific patient. Features were arranged along the y axis based on their importance, which was given by the mean of their absolute Shapley values. The higher the feature was positioned in the plot, the more important it was for the model

Figure 4A, B and Table 3 showed the performance of each ML model on the training set and validation set. Figure 4C showed the AUC score forest plot of each model; Fig. 4D showed the reliability curve of each model. XGBoost had the best performance both in the training set (AUROC: 0.781, 95%CI: 0.772–0.791) and the validation set (AUROC: 0.778, 0.758–0.798). Figure 5 showed the optimization process of the XGBoost model (Fig. 5A, Fig. 5B and Fig. 5D displayed the ROC curve of the train, validation and test of the XGBoost model by 10-fold cross-validation. Figure 5C showed the learning curve of the XGBoost classifier. Figure 5E showed the reliability curve of XGBoost model). After 10-fold cross-validation, the AUROC value of the model on the training set was 0.809 (95%CI: 0.800–0.818), and the AUROC value on the validation set is 0.772 (95%CI: 0.743–0.800), and the AUROC value on the test set is 0.751 (95% CI: 0.731–0.770). At this point the model has the best predictive stability and accuracy. Given that the performance of the model on the validation set as evaluated using the AUROC index did not exceed that on the test set or the exceed ratio was less than 10%, the fitting can be considered as successful, and XGBoost model can be used for classification of the future datasets.

**Table 3:** Predictive performance comparison of the 5 types of machine learning classifiers in the training set and the validation set, (Mean ± SD)

ML classifiers	Accuracy(95%CI)	Accuracy(95%CI)	Sensitivity(95%CI)	Specificity(95%CI)	NPV (95%CI)
**Training sets**					
**XGBoost**	0.782(0.772–0.792)^a^	0.682(0.666–0.698)	0.746(0.726–0.767)	0.671(0.649–0.692)	0.957(0.955–0.959)
**RF**	0.679(0.635–0.724)	0.679(0.635–0.724)	0.748(0.713–0.783)	0.640(0.595–0.684)	0.953(0.945–0.961)
**AdaBoost**	0.648(0.612–0.685)	0.648(0.612–0.685)	0.785(0.735–0.835)	0.590(0.544–0.636)	0.953(0.945–0.962)
**GNB**	0.662(0.655–0.669)	0.662(0.655–0.669)	0.692(0.658–0.726)	0.683(0.645–0.722)	0.953(0.949–0.956)
**MLP**	0.683(0.670–0.695)	0.683(0.670–0.695)	0.711(0.658–0.764)	0.683(0.621–0.746)	0.953(0.948–0.958)
**Validation sets**					
**XGBoost**	0.777(0.757–0.797)^a^	0.678(0.663–0.694)	0.748(0.717–0.780)	0.660(0.614–0.706)	0.956(0949–0.962)
**RF**	0.679(0.635–0.724)	0.679(0.635–0.724)	0.748(0.713–0.783)	0.640(0.595–0.684)	0.953(0.945–0.961)
**AdaBoost**	0.648(0.612–0.685)	0.648(0.612–0.685)	0.785(0.735–0.835)	0.590(0.544–0.636)	0.953(0.945–0.962)
**GNB**	0.662(0.655–0.669)	0.662(0.655–0.669)	0.692(0.658–0.726)	0.683(0.645–0.722)	0.953(0.949–0.956)
**MLP**	0.683(0.670–0.695)	0.683(0.670–0.695)	0.711(0.658–0.764)	0.683(0.621–0.746)	0.953(0.948–0.958)

*Note*: ^a^indicated that the best performance of the ML classifier in the training set and validation sets was XGBoost (Ranked according to AUC)

*Abbreviation*:*ML* Machine learning, *XGBoost* Extreme gradient boosting, *RF* Random forest, *AdaBoost* Adaptive boosting, *GNB* Gaussian naive Bayes, *MLP* Multilayer perceptron, *AUROC* Area under the receiver operating characteristic curve, *NPV* Negative predictive value, *CI* Confidence interval

Summary plots for SHAP values was shown in Fig. 5F. For each feature, one point corresponded to a single patient. A point’s position along the x axis represented the impact that feature had on the model’s output [23]. The feature of ETE had the largest SHAP value. It was the highest risk factor for the LNM. While features were arranged along the y axis based on their importance on the model, The higher the feature was positioned the more important it was for the model. It was evident that the most important factor for the model was the tumor size and the least one was race.

We optimized the XGBoost algorithm through 10-fold cross-validation, and its best performance on the training set AUROC value (AUC: 0.809, 95%CI: 0.800–0.818) is better than the traditional LR model (AUC: 0.780, 95%CI: 0.772–0.787).

## Discussion

The clinical strategy for patients with PTMC remained controversial. Should we opt for surgery or just active surveillance only? If surgery was being performed, would it necessary to do the bilateral lobar resection (BLR), or only operating on the affected lobe (unilateral lobectomy)? For non-invasive, clinically node-negative (cN0) PTMC, would it have a better prognosis if the prophylactic central lymph node dissection (pCLND) was carried out? The availability of suitable prognostic models became important. Conventional LR model and ML each had their own advances and limitations. We established models for cervical LNM by conventional LR and ML algorithms with the goal of evaluating their performance in prediction of LNM in PTMC.

### Risk factors for cervical LNM of PTMC

In the ML model and the traditional regression model analyses, four risk factors were found to be most closely associated with cervical LNM in PTMC. These includes, extrathyroidal metastasis (ETE), tumor size, age, and multifocality. Among them, ETE was the most important factor affecting the outcomes predicted by the models. It was confirmed by many clinical research that ETE predicts negative clinical outcomes in papillary thyroid cancer [24]. All levels of extrathyroidal extension, including microscopic, were associated with a increased risk for nodal and distant metastasis [25]. Unfortunately, minimal ETE was often difficult to identify before the operation, The utility of intra-operative frozen section for the evaluation of microscopic extrathyroidal extension in papillary thyroid carcinoma seemed important and the patients who were diagnosed as ETE in postoperative pathology without pCLND. might be recommended to be intensively followed up. Since tumor size was a simple parameter that can be determined with ultrasound images, it was widely used to predict the aggressiveness of PTMC to aid clinical decision-making. In PTC, the larger the tumor was, the greater the risk of cervical LNM was [26], Similar results were found in PTMC [27]. In our study, patients with tumors ≥5 mm had a significantly higher risk of cervical LNM compared to those with tumors < 5 mm, which was consistent with the results of previous clinical studies and meta-analyses [24]. Age was an important risk factor for thyroid cancer. Children and young adults often present aggressive disease patterns and advanced stages, and had a relatively high rate of PTMC lymph node metastasis [28]. Thus, we put children and young adults into the same category (< 25 years old) with a risk value of 1. Compared to this group，for adults, with every 5 years of age increasing, the risk of PTMC cervical lymph node metastasis gradually decreased. The OR values were: 0.586, 0.362, 0.26, and 0.239 showing a relatively obvious and gradual decreasing trend. Therefore, for older patients, the extent of surgery should be conservative and less frequent follow-up was allowed. On the other hand, younger patients, especially children and adolescents, should be treated more aggressively and followed up closely to reduce the risk of recurrence. The multifocality of the tumor was also one of the factors closely related to PTMC cervical lymph node metastasis. Papillary thyroid carcinoma often occurs in the form of two or more independent lesions in the thyroid (18–87%) [29]. Multifocality may arise from intrathyroidal metastases from a single malignant lesion or from multiple lesions of independent origin with intrathyroidal metastases [30]. In our study, the incidence of multifocal PTMC was 35.05%. It was a major risk factor in both the ML and the conventional LR models. The results were consistent with previous studies [31]. In contrast, although some previous studies had suggested that bilateral tumor was a risk factor for thyroid neck lymph node metastasis [29]. Our study found that laterality was not associated with cervical lymph node metastasis. There were no significant differences between unilateral or bilateral tumors in either univariate or multivariate analysis. Histopathological subtypes were also closely linked to the lymph nodes of PTMC. Some histological subtypes of PTC were classified as aggressive variants of PTC (AVPTC), which included columnar/tall-cell variant (TCV), and diffuse sclerosis subtype [32]. In our study, results consistent with previous studies were also obtained. However, among the above risk factors of cervical LNM, histiocytic subtype was a factor that could not be determined before surgery. ETE and multifocality were often hard to find during preoperative routine inspection. They largely depended on the accuracy of intraoperative frozen section pathology analysis. This may limit the applicability of the machine learning model in preoperative clinical prediction.

The relationship between tumor prognosis and population sociology has received increasing attention. In our study, in addition to age, patient gender was also found to be associated with cervical LNM in PTMC. It was well known that the prevalence of thyroid cancer in men was much lower than that in women, although men had a higher rate of cervical LNM and a poorer prognosis [33]. In our study, we found that male sex was also a risk factor for cervical LNM. Compared with women, the OR was 2.279. The mechanism was unclear, although some studies suggested that estrogen might regulate the proliferation of thyroid cells by combining with estrogen receptor (ER) α and ERβ [34]. Since ER expression levels differ between males and females, this may be one of the reasons for the difference in the sex ratio of cervical LNM in PTMC patients. Race and region of residence were also associated with the risk of cervical LNM in PTMC. Black race was a protective factor for cervical LNM (OR: 0.551) relative to white race, while other races were not significantly different from white race. In a previous study on race and PTC prognosis in the SEER database, black Americans had lower overall survival than white Americans (HR: 1.127). However, there were fewer lymph node metastases in classic papillary thyroid carcinoma (OR: 0.476) and follicular subtype papillary thyroid carcinoma (OR: 0.522) in black Americans [35]. Genetic variation may be a possible mechanism for the differences. In addition, it is possible that the limitation in health care resources for black Americans might have leaded to less prophylactic neck dissections and/or less proper ultrasounds, causing an overt reduction in observed incidence rate. The distribution of PTMC varied by region in the United States. The Pacific coast accounts for the largest proportion (42.767%) and had the highest incidence of lymph node metastasis. (11.9%). This may be related to the uneven iodine intake and different racial distribution of residents. However the factors that effected the cancer disparities are complex, including lifestyle, income, health security and access to affordable health services of high quality [36]. One of the thought-provoking results in our study was the effect of marital status on cervical LNM in PTMC. We found that being single was the only marital- status related risk factor for the cervical LNM. Even the divorced, widowed or separated had a better outcome than the single. It is possible that spouses may encourage patients to seek medical attention for alarming symptoms thus resulting an early diagnosis of the tumor [37, 38]. In addition, the support of the family especially the spouse might help reduce the stress and depression in the patients which might help recovery from the disease. Our analysis showed that social and family relationship was an important factor affecting tumor prognosis.

### Predictive model performance comparison

There were several ML algorithms commonly used in predictive model construction. Different ML approaches had different advantages and disadvantages. In this study, compared with the other four ML algorithms (AdaBoost, RF, GNB and MLP), XGboost was found to be the best model for predicting cervical LNM in PTMC using a dataset derived from the SEER database. In this study, we found the XGboost algorithm performance best both in AUC and in the accuracy of model construction. Its accuracy was improved in optimized procession.

Compared with conventional LR model, machine learning methods performance better in predicting cervical LNM outcomes. Though the advantage in AUC value was not so obvious. This may be attributable to the variables that were selected into the model. There were no correlation or collinearity among all variables. In addition, the variables selected were simple and there were not so many features. The fact that the machine learning method show only limited advantage in our study indicates if the variables were simple and did not have any collinearity, conventional LR could also be a good choice for model construction. It was likely that only when there were complex, high-dimensional data available the ML method might show much more substantial advantages.

### Visualization of feature importance

We visualize OR and 95%CI for variables identified by conventional LR in the form of nomogram and forest plot map to help understand the model [39]. Nomogram and forest plot clearly showed the ETE, larger tumor size, histology of column cell, multifocality, male, single, and the region of Pacific Coast were all risk factors while older age, race of black and the histology of follicular variant and encapsulated were protective factors. ETE had the biggest OR ration. It was a very important positive risk factor in predicting the result of the cervical LNM in PTMC. In contrast, the feature of marital status and race were not as important as other factors.

For a long time the ML only provided a ranking of feature importance and did not specify whether each important factors was protective or dangerous in the way LR did. The “black-box” characteristics of ML algorithms made it difficult to understand. In this study we leverage SHAP to illustrate the factors in the predict model constructed [23]. The map provided by SHAP helped to visualize the prediction power of valuables. With SHAP of XGboost, we can easily find that ETE, gender, multifocality and tumor size were clearly the positive risk factors for the cervical LNM. ETE was the most important risk factor of the cervical LNM. This agreed with what was shown by nomogram and forest plot from LR modeling.

The main limitations of this study were as follows: First, this study was mainly limited by the retrospective nature of the analysis, so confounding is inevitable. Second, the identification of cervical LNM was primarily derived from the collection of data on cases where therapeutic lymph node dissection was performed. The incidence of cervical LNM in this study was much lower than it was in some other studies. This suggested that the incidence of cervical LNM may be underestimated. In addition, we did not differentiate the central and lateral LNM of cervical LNM. There may be different characteristics of these two kinds of LNM which is also important for clinical strategy. Third, SEER database only included patients lived in the United States of America. The factor of residence in our model might only be representative of this particular cohort and reflected health care differences, such as access to proper prophylactic neck dissections and/or ultrasounds, existing in different geological locations in America. It was possible that the residence factor was either not relevant or contributed in a different way in other populations of the world. Fourth, some high-risk factors or characteristics associated with cervical LNM were not documented in the SEER database, such as autoimmune thyroid disease (AITD), preoperative ultrasound, imaging, fine-needle biopsy, or molecular analysis. We hoped that in the future, prospective multicenter studies with long-term follow-up data will help obtain additional useful clinical or social characteristics to further improve the model.

## Conclusions

ETE, tumor size, multifocality and age were the most important risk features for the model. This argues that young patients, or patients with tumors that are multifocal, or ETE, or size ≥5 mm be followed up closely.

In this study we found machine learning algorithm offering improvement over traditional LR in predicting cervical LNM of PTMC. We verified its utility in the specific use case and demonstrated its value in helping the clinicians make the right decision. We believe that with its strong data processing ability and learning ability, ML will become a promising predictive tool when large, complex data become available.

## Supplementary Information

**Additional file 1: Table S1**. Definition and classification of risk factors for cervical lymph node metastasis in PTMC.

## Data Availability

The data used to support the findings of this study are available from the SEER database (seer.cancer.gov).

## Abbreviations

LNM: lymph node metastasis
PTMC: papillary thyroid microcarcinoma
PTC: papillary thyroid carcinoma
ML: machine learning
XGBoost: extreme gradient boosting
RF: random forest
AdaBoost: adaptive boosting
GNB: gaussian naive bayes
MLP: multi-layer perceptron
LR: logistic regression
AUROC: area under the receiver operating characteristic
ETE: extrathyroidal extension
BLR: bilateral lobar resection
pCLND: prophylactic central lymph node dissection
ER: estrogen receptor
SHAP: SHapley Additive exPlanations
AVPTC: aggressive variants of papillary thyroid carcinoma
TCV: columnar/tall-cell variant
GDBT: Gradient Boosting Decision Tree
AITD: autoimmune thyroid disease
