# Supplementary material for: Exploring risk factors for cervical lymph node metastasis in papillary thyroid microcarcinoma: construction of a novel population-based predictive model
Source: BMC Endocr Disord. 2022 Nov 4;22:269. doi: 10.1186/s12902-022-01186-1 (PMC9635156; doi:10.1186/s12902-022-01186-1)
Supplement: Supplementary file 1 — Additional file 1: Table S1. Definition and classification of risk factors for cervical lymph node metastasis in PTMC. [file 12902_2022_1186_MOESM1_ESM.docx]

**Table S1**. **Definition and classification of risk factors for cervical lymph node metastasis in PTMC**

| **Variables** | **Classification** | **Assignment** |
| --- | --- | --- |
| Cervical LMN | 0~1 | 0: No; 1:Yes |
| Age | 0~4 | 0: ＜25; 1: 25-39; 2: 40-54; 3: 55~69; 4: ≥70 |
| Gender | 0~1 | 0: Female; 1: Male |
| Race | 0~4 | 0: White; 1: Black; 2: Others* |
| Region | 0~3 | 0: East; 1: Pacific Coast; 2: Northern Plains; 3: Southwest; |
| Marital status | 0~3 | 0: Married; 1: Single; 2: Divorced; 3: Widowed; |
| Histology^#^ | 0~5 | 0: Papillary adenocarcinoma; 1: Papillary carcinoma, follicular variant; 2: Papillary carcinoma, oxyphilic cell ; 3: Papillary carcinoma, encapsulated ; 4: Papillary carcinoma, columnar cell; 5: Nonencapsulated sclerosing carcinoma |
| Tumor size^##^ , | 0~1 | 0: ＜5mm; 1: ≥5mm |
| Extrathyroidal extension** | 0~1 | 0: No; 1: Yes |
| Multifocality | 0~1 | 0: Solitary tumor; 1: Multifocal tumor |
| Laterality | 0~1 | 0: Unilateral; 1: Bilateral |

**Note**:

*Others include American Indian, Alaska Native, Asian or Pacific Islander;

^#^Histology was performed according to the Third Edition of International Classification of Disease for Oncology [ICD-O-3] code: 8260/3: Papillary adenocarcinoma; 8340/3: Papillary carcinoma, follicular variant; 8342/3: Papillary carcinoma, oxyphilic cell; 8343/3: Papillary carcinoma, encapsulated; 8344/3: Papillary carcinoma, columnar cell; 8350/3: Nonencapsulated sclerosing carcinoma.

Tumor size^##^ is defined as the maximum diameter of the tumor.

Extrathyroidal extension (ETE)** is defined as extension of the primary tumor beyond the thyroid capsule into the perithyroidal soft tissues, strap muscles, and adjacent structures.

**Abbreviations**: PTMC: Papillary thyroid microcarcinoma; cervical LNM: Cervical lymph node metastasis
